# Mechanism of vaccinia viral protein B14–mediated inhibition of IκB kinase β activation

**DOI:** 10.1074/jbc.RA118.002817

**Published:** 2018-05-10

**Authors:** Qingyu Tang, Sayan Chakraborty, Guozhou Xu

**Affiliations:** From the Department of Molecular and Structural Biochemistry, College of Agriculture and Life Sciences, North Carolina State University, Raleigh, North Carolina 27695

**Keywords:** molecular docking, autophosphorylation, protein kinase, virus, inhibition mechanism, B14, IKKβ, protein kinase, trans autophosphorylation, vaccinia virus

## Abstract

Activation of IκB kinase β (IKKβ) is a central event in the NF-κB–mediated canonical pro-inflammatory pathway. Numerous studies have reported that oligomerization-mediated trans autophosphorylation of IKKβ is indispensable for its phosphorylation, leading to its activation and IKKβ-mediated phosphorylation of substrates such as IκB proteins. Moreover, IKKβ's interaction with the NF-κB essential modifier (NEMO) is necessary for IKKβ activation. Interestingly, some viruses encode virulence factors that target IKKβ to inhibit NF-κB–mediated antiviral immune responses. One of these factors is the vaccinia viral protein B14, which directly interacts with and inhibits IKKβ. Here we mapped the interaction interface on the B14 and IKKβ proteins. We observed that B14 binds to the junction of the kinase domain (KD) and scaffold and dimerization domain (SDD) of IKKβ. Molecular docking analyses identified key interface residues in both IKKβ and B14 that were further confirmed by mutational studies to promote binding of the two proteins. During trans autophosphorylation of protein kinases in the IKK complex, the activation segments of neighboring kinases need to transiently interact with each other's active sites, and we found that the B14–IKKβ interaction sterically hinders direct contact between the kinase domains of IKKβ in the IKK complex, containing IKKβ, IKKα, and NEMO in human cells. We conclude that binding of B14 to IKKβ prevents IKKβ trans autophosphorylation and activation, thereby inhibiting NF-κB signaling. Our study provides critical structural and mechanistic information for the design of potential therapeutic agents to target IKKβ activation for the management of inflammatory disorders.

## Introduction

The inhibitor of κB kinase (IKK)^2^ complex plays a central role in regulating the activation of NF-κB transcription factors, which are the master regulators of a variety of cellular processes, particularly immune and inflammatory responses (1). Although many different inducers activate NF-κB, most of the signaling pathways converge on activation of IKK (2). IKK is a large protein complex that comprises three major subunits, the kinase subunits IKKα (IKK1) and IKKβ (IKK2) and a regulatory subunit known as NF-κB essential modifier (NEMO, also called IKKγ). IKKβ harbors an N-terminal kinase domain (KD), an ubiquitin-like domain (ULD), a scaffold and dimerization domain (SDD), and a C-terminal NEMO-binding domain (NBD). It contributes to the majority of the IκB kinase activity of IKK and plays a dominant role in the canonical NF-κB pathway by phosphorylating IκBα. IKKα shares more than 50% sequence identity with IKKβ and plays an indispensable role in the noncanonical NF-κB pathway (3, 4).

The main substrates of IKK are the IκB family of proteins, which are the inhibitory proteins of NF-κB (5). There are at least nine IκB members in the human genome, three of which (IκBα, IκBβ, and IκBϵ) are classical IκB proteins functioning as NF-κB inhibitors (6). The most extensively studied IκB member, IκBα, contains two serine residues in the destruction motif consensus DSGψ*X*S (ψ stands for hydrophobic residue, *X* is any residue) in its N-terminal region. These two serine residues serve as the IKKβ phosphorylation sites. The doubly phosphorylated destruction motif is then recognized by the β-TrCP–SCF ubiquitin ligase for subsequent ubiquitination and degradation. After degradation of IκBα, NF-κB is then released into the nucleus to regulate gene transcription (5, 7).

Since its discovery in 1996, the function of the IKK complex has been under intensive investigation. However, its mechanism of activation remains elusive. In response to upstream signaling cues such as pro-inflammatory stimuli, the activation segments of IKKβ are phosphorylated at Ser-177 and Ser-181 (7–9). In mouse embryonic fibroblasts, some upstream kinases, such as transforming growth factor β–activated kinase 1 (TAK1), have been shown to be required for IKKβ activation. However, TAK1 only phosphorylates IKKβ at Ser-177, which is a priming event that enables IKKβ to activate itself by phosphorylating Ser-181 (10). Other lines of evidence have pointed out that oligomerization-mediated trans autoactivation is required for IKKβ activation. It has been found that the overexpressed and reconstituted IKK complex is active (11, 12) and that binding to different types of poly-ubiquitin chains via NEMO can activate the IKK (13, 14). Indeed, in NEMO knockout cells, IKK activity is completely abolished (15). It is possible that activation of IKKβ requires its interaction with NEMO, which, in turn, probably facilitates its oligomerization and trans autophosphorylation events (3, 7). Our previous structural studies of IKKβ have indicated that the kinase domains in the IKKβ dimer face away from each other so that they are unable to trans phosphorylate each other (7). Therefore, larger oligomeric forms must exist so that the trans autophosphorylation event can take place.

NF-κB–dependent gene expression is essential for stimulating the pro-inflammatory and immune responses to defend viral infections, and viruses such as vaccinia virus have, accordingly, evolved strategies to escape from the NF-κB–dependent anti-viral immune response by inhibiting the activation of IKK (16). VACV encodes a 17-kDa protein, B14, that interacts directly with IKKβ to inhibit its activation, consequently blocking NF-κB activation (17). The viral protein blocks phosphorylation of the activation segment of IKKβ to impede its activation but does not inhibit its kinase activity, as it has been shown to be incapable of inhibiting a constitutively active mutant form of IKKβ (S177E/S181E) (18). Interestingly, binding of B14 does not interfere with the assembly of the IKK holocomplex (19). In this study, we set out to elucidate the mechanism of VACV B14-mediated binding and inhibition of IKKβ activation. Study of this interaction has enlightened us regarding the mechanism of IKKβ activation from the perspective of a viral inhibitor. Dysregulation of the IKK/NF-κB pathway is associated with numerous diseases, such as diabetes, cancer, and inflammatory and autoimmune diseases (20). Therefore, our study has provided critical structural and mechanistic information for the future design of new therapeutic agents to specifically target IKKβ activation for the treatment of these devastating human diseases.

## Results

### B14 inhibits IKKβ activation but not its kinase activity

**Figure 1.**
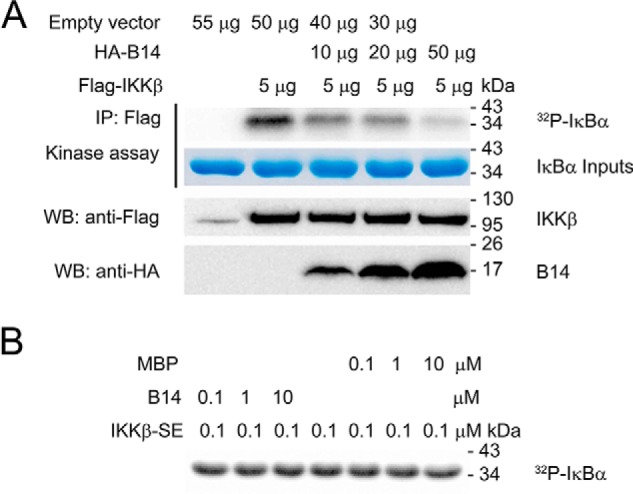
**The VACV protein B14 inhibits IKKβ activation but cannot impede its IκBα kinase activity.** *A*, to elucidate the role of B14-mediated inhibition of IKKβ activation, we analyzed the kinase activity of autoactivated IKKβ in HEK293T cells co-transfected with full-length human IKKβ and varying amounts of B14 construct. The empty vector pcDNA3 was used to adjust the total DNA of 55 μg used per transfection. *IP*, immunoprecipitation; *WB*, Western blot. *B*, to rule out the possibility that B14 also inhibits the activated IKKβ with dual phosphorylation on Ser-177 and Ser-181 in the phosphorylation of IκBα, *in vitro* kinase assays with the constitutively active IKKβ-S177E/S181E (*IKK*β-*SE*) and B14 proteins were conducted. Maltose binding protein (*MBP*) was used as a negative control. Each experiment was repeated three times.

Previous studies have shown that B14 inhibits IKKβ phosphorylation on Ser-177 and Ser-181 of its activation segment (18). In this study, we used the HEK293T cell overexpression system to examine human IKKβ autoactivation by assessing its phosphorylation on IκBα. To elucidate the role of B14-mediated inhibition of IKKβ activation, we co-transfected HEK293T cells with both FLAG-IKKβ and HA-B14. IKKβ protein was then immunoprecipitated with anti-FLAG antibody, and its kinase activity was assessed in kinase assay with [γ-^32^P]ATP. The purified recombinant human IκBα protein was used as a substrate of IKKβ. Consistent with other studies (17, 18), the viral protein B14 significantly inhibits IKKβ autoactivation in a dose-dependent manner (Fig. 1*A*). Because varying amounts of B14 construct were co-transfected with the same amount of WT IKKβ construct, the inhibitory effect of B14 on IKKβ autoactivation can be manifested by its loss of activity on IκBα phosphorylation. To rule out the possibility that B14 also inhibits the activated IKKβ with dual phosphorylation on Ser-177 and Ser-181 in the phosphorylation of IκBα, we conducted an *in vitro* kinase assay with the purified recombinant IKKβ-SSEE (constitutively active S177E/S181E mutant). B14 did not show any distinguishable inhibitory effect on IKKβ kinase activity (Fig. 1*B*), confirming that it does not inhibit the active IKKβ.

### B14 binds both the KD and SDD of IKKβ

**Figure 2.**
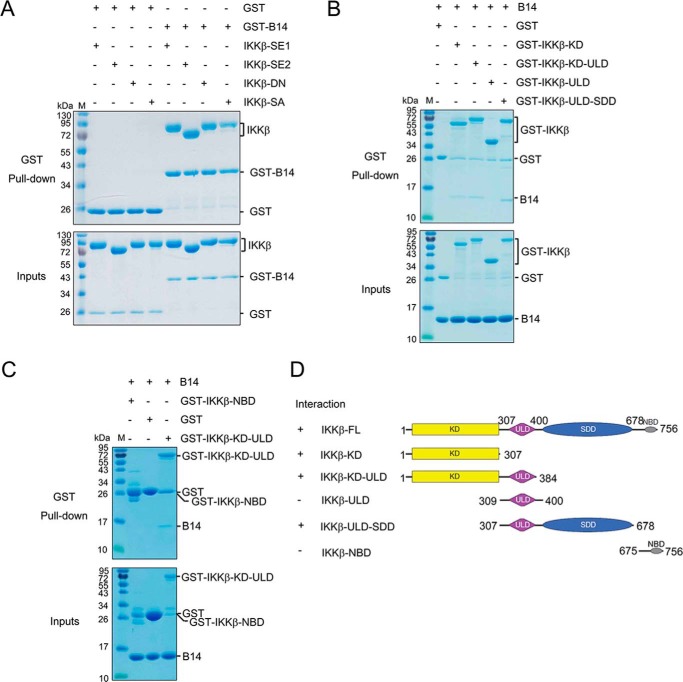
**Map of the B14 interaction domains on human IKKβ**. *A*, pulldown of IKKβ by GST-B14. GST protein alone was used as a negative control. *IKK*β*-SE1*, IKKβ (1–756) S177E/S181E; *IKK*β*-SE2*, IKKβ (1–678) S177E/S181E; *IKK*β*-DN*, IKKβ (1–756) D145N; *IKK*β*-SA*, IKKβ (1–756) S177A/S181A. *B* and *C*, pulldown of B14 by GST-IKKβ truncation mutant proteins. *D*, domain boundaries of the IKKβ constructs are shown on the *right*, and the binding property between each IKKβ construct and B14 is summarized on the *left*. Each experiment was repeated three times. *M* stands for protein molecular weight standard marker.

In the structure of IKKβ, the KD, ULD, and SDD interact with each other to form an integral trimodular structural unit; however, the NBD has been shown to be an independently folded domain connected with a flexible linker to the rest of the protein (21). To map the B14 binding region on IKKβ, we used a GSH S-transferase fusion B14 protein (GST-B14) to pull down the purified human IKKβ proteins (Fig. 2*A*). B14 binds to both full-length IKKβ and a construct without the NBD (4–675), indicating that the NBD is not required for B14–IKKβ interaction. In addition, B14 binds to both the constitutively active IKKβ-SSEE and inactive IKKβ-D145N, showing that its activation state does not affect B14 association. Furthermore, Ser-to-Glu or Ser-to-Ala substitutions on Ser-177 and Ser-181 of the activation segment do not influence B14 binding, suggesting that the binding may not engage the kinase activation segment.

To determine the B14-interacting domains on IKKβ, we expressed the GST fusion constructs of the KD (residues 1–307), KD-ULD (residues 1–384), ULD (residues 309–400), and ULD-SDD (residues 307–678) of human IKKβ in *Escherichia coli* (Fig. 2, *B* and *D*). The recombinant GST-SDD constructs were not soluble, indicating that, when expressed alone separately, the SDD domain cannot be folded properly. We used the GST-IKKβ fusion proteins to pull down untagged B14 protein. All constructs pulled down B14, except GST-ULD, which clearly demonstrates that the ULD is not required for B14 binding to IKKβ. GST alone did not bind B14, indicating that the binding between B14 and other IKKβ constructs is specific (Fig. 2*B*). The NBD of IKKβ does not contribute to IKKβ binding to B14 because the GST-NBD (residues 675–756) IKKβ construct did not pull down B14 (Fig. 2*C*). Based on the results, we can conclude that B14 interacts with IKKβ on the junction between KD and SDD domains.

### The M2 and M4 regions of IKKβ are required for its interaction with B14

**Figure 3.**
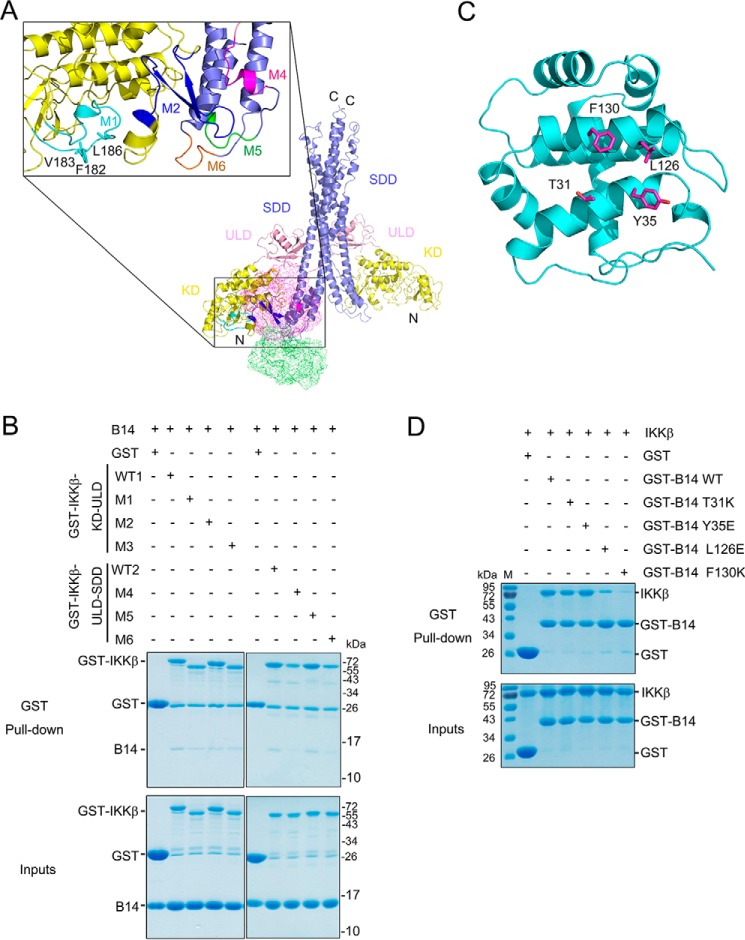
**The M2 and M4 segments of IKKβ are required for B14 binding.** *A*, molecular docking models of IKKβ–B14 interaction. The KD, ULD, and SDD of human IKKβ (PDB ID 4E3C) are colored *yellow*, *pink*, and *slate*, respectively. The potential B14-interacting segments present on IKKβ protein are shown as *M1*, *M2*, and *M4-M6* and colored *cyan*, *blue*, *magenta*, *green*, and *orange*, respectively. Three residues, Phe-182, Val-183, and Leu-186 on the M3 segment, are shown in *stick representation* and colored *cyan. B*, pulldown of B14 by GST-IKKβ mutants. GST protein alone was used as a negative control. GST-IKKβ-KD-ULD contains residues 1–384 of human IKKβ, and GST-IKKβ-ULD-SDD harbors residues 307–678 of human IKKβ, both of which are used as the WT positive controls (*WT1* and *WT2*). In addition, the M1 mutant has residues 179–197substituted with GGGGSGGS, M2 has residues 235–260 replaced with GGGGSGGS, M3 contains F182K/V183K/L186K mutations, M4 has residues 408–416 replaced with GGGGS, M5 has residues 421–426 substituted with GGGGS, and M6 has residues 577–583 replaced with GGGGS. *C*, the B14 structure is represented as a *ribbon diagram* and is colored *cyan*, with the potential IKKβ interacting residues shown as *stick representation* and colored *magenta. D*, pulldown of human IKKβ (1–675, S177E/S181E) by GST-B14 mutants. All experiments were repeated three times and yielded similar results. *M* stands for protein molecular weight standard marker.

Because the crystal structures of both the VACV B14 and IKKβ proteins are available, we conducted computational molecular docking to reconstruct the B14–IKKβ interaction with FRODOCK (22, 23). In parallel, we also used CLUSPRO (24, 25) to search for the binding interface, where the surface of the KD and SDD junction of IKKβ was chosen as a space restraint. We have included both the *Xenopus* IKKβ structure (PDB code 3QA8) in a closed conformation and a human IKKβ structure (PDB code 4E3C) in an open conformation for the modeling. We identified the same potential binding surface composed of both KD and SDD of both orthologous IKKβ structures. Five surface regions of IKKβ, encompassing residues ranging from 179–197 (M1), 235–260 (M2), 408–416 (M4), 421–426 (M5), and 577–583 (M6) are contacting B14 in the docking models (Fig. 3*A*). To verify the docking models, we have substituted all residues of the M1 and M2 segments with Gly or Ser, mutated three key interacting residues (F182K, V183K, and L186K) of M3 on the GST-KD-ULD construct, and changed all residues on the M4, M5, and M6 segments to Gly or Ser on the GST-ULD-SDD construct. The GST fusion human IKKβ mutant constructs were then used to pull down the untagged B14 protein. We found that the M2 and M4 mutants have an almost 60% lower binding affinity compared with the WT constructs. The M6 mutant has a light decrease in its interaction with B14, whereas the M1, M3, and M5 mutants have no significant changes in B14 binding (Fig. 3*B* and Fig. S1).

Based on the molecular docking modeling, the residues Thr-31, Tyr-35, Leu-126, and Phe-130 present on the surface of B14 can potentially contact IKKβ (Fig. 3*C*). Phe-130 of B14 has been demonstrated previously to be required for IKKβ binding and inhibition (19) and, in turn, it validates our modeling results. To test which of the residues contribute to B14 interaction with IKKβ, we created single-substitution mutant constructs of GST-B14 to pull down the full-length human IKKβ protein (Fig. 3*D* and Fig. S1*C*). Both the mutants T31K and Y35E demonstrated a significant decrease in binding affinity as the WT B14 to IKKβ; L126E had a slight loss of binding, whereas F130K had negligible binding to IKKβ. This is consistent with a previous study showing that the B14 F130K mutant protein not only lost its binding to IKKβ but was also unable to inhibit NF-κB signaling (19). We postulate that Phe-130 and Leu-126 of B14 are likely to create a small hydrophobic surface patch to bind the KD and SDD junction surface of IKKβ in forming the inhibitory complex.

### Binding of B14 to IKKβ does not block the kinase activation segment

**Figure 4.**
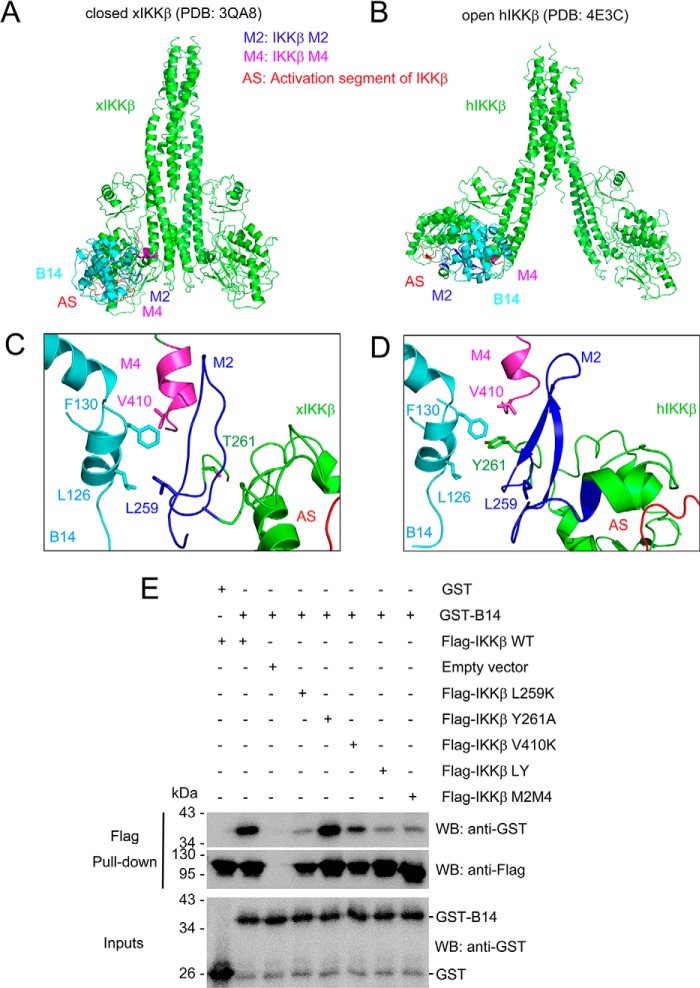
**B14-IKKβ binding interface.** *A* and *B*, models of B14/IKKβ complex structures. Closed *Xenopus* IKKβ (xIKKβ, PDB code 3QA8, *A*) and open human IKKβ (hIKKβ, PDB code 4E3C, *B*) structures are shown as *ribbons* and colored *green*. VACV B14 is shown as a ribbon and colored *cyan*. M2, M4, and the activation segment of IKKβ (*AS*) are colored *blue*, *magenta*, and *red*, respectively. *C* and *D*, B14-IKKβ binding interface for xIKKβ and hIKKβ, respectively. The key interacting residues are shown as *sticks. E*, GST-B14 was pulled down by human WT and mutant IKKβ. The empty vector was expressed as a control. *LY* refers to the L259K and Y261A IKKβ double mutant. The M2M4 IKKβ mutant contains both the M2 and M4 substitutions described in Fig. 3. *WB*, Western blot. The experiment was repeated three times with consistent results.

We used the identified interface to perform a local search with a molecular docking program, ROSETTA (26, 27). We also included both the *Xenopus* IKKβ structure (PDB code 3QA8) in a closed conformation and a human IKKβ structure (PDB code 4E3C) in an open conformation for the modeling to indicate the evolutionarily conserved B14 binding. In these refined binding models, the small hydrophobic patch centered on Phe-130 of B14 interacts with both the M2 and M4 segments of IKKβ, which has no contact with the activation segment of the kinase domain (Fig. 4, *A–D*). To verify this predicted binding interface, we created six full-length human IKKβ mutants with the substitutions L259K, Y261A, V410K, L259K/Y261A, and L259K/Y261A/V410K, and the M2M4 mutant. These mutants were expressed in human kidney embryonic 293F (HEK293F) cells with an engineered N-terminal FLAG tag. The mutant recombinant proteins were used to pull down purified GST fusion B14 protein expressed in *E. coli*. The IKKβ L259K/Y261A/V410K mutant was not expressed in HEK293F cells, indicating that substituting all three residues disturbs its proper folding and stability. The L259K and L259K/Y261A mutations severely weakened IKKβ binding to B14, whereas V410K or Y261A alone had a minor effect on their interaction (Fig. 4*E*). Tyr-261 is not conserved in *Xenopus* IKKβ (Fig. 4*C*), indicating that Tyr-261 is not critical. The M2M4 mutant has a similar binding pattern to B14 as L259K/Y261A. This shows that the three chosen residues in the M2M4 regions account for most of the binding affinity for B14. In this confirmed binding model of both the closed and open IKKβ structures, the activation segment of the kinase domain is exposed for substrate binding, which explains why B14 does not directly inhibit IKKβ kinase activity.

### A model for B14-mediated inhibition of IKKβ activation

**Figure 5.**
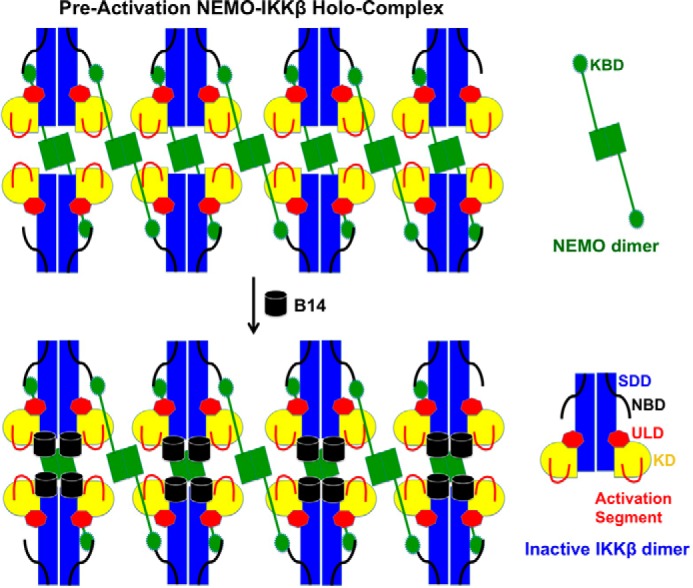
**A model for B14-mediated inhibition of IKKβ activation.** The KBD of NEMO interacts with the NBD of IKKβ, which cross-links IKKβ to a large oligomer. Upon activation by upstream signaling events or by high IKK concentration, the activation segments of the neighboring KDs can contact each other for trans autophosphorylation. Binding of B14 to the junction of KD and SDD of IKKβ causes a steric hindrance that impedes the optimal contact between KDs in the IKK complex, which, in turn, blocks the insertion of the activation segment of one KD to the active site of another for trans autophosphorylation and activation.

Our previous measurement of the recombinant full-length human NEMO and IKKβ proteins by size exclusion chromatography with multiangle light scattering has shown their dimeric status in solution. The measured molecular mass of the reconstituted IKK complex is around 2000 kDa, which corresponds to ∼12–16 NEMO and IKKβ molecules in each complex assembly (28). Based on this study, we postulate that, instead of one NEMO binding to one IKKβ molecule to form a heterodimer, NEMO uses each of its two kinase binging domains to interact with an NBD in two separate IKKβ molecules. In this mode of alternating binding, the NEMO protein cross-links IKKβ to form a high-order oligomer in which trans autophosphorylation and activation can occur (Fig. 5 and Fig. S2*B*).

The crystal structures of the IKKβ dimer have distinct closed and open conformations (7, 29, 30). There is a certain degree of flexibility in the SDD domain so that the KD and the proximal region of SDD can swing toward or away to adopt either a closed or an open dimer conformation (Fig. S2). In the *Xenopus* IKKβ structure, although it is an S177E/S181E constitutively active mutant, the presence of a specific kinase inhibitor renders it in an inactive kinase form, and IKKβ adopts a closed dimer conformation (7). Surprisingly, the active human IKKβ is in a unique open dimer conformation (29, 30). The IKKβ structure may vary between multiple transitional conformations in solution, as proposed previously, or probably the closed conformation represents the inactive state, whereas the open conformation indicates the active state.

We hypothesize that the IKKβ undergoes at least two transitional stages during its activation, a preactivation complex and a postactivation (activated) complex. In the preactivation complex, the activation segments of the neighboring KDs can contact each other for trans autophosphorylation (Fig. 5 and Fig. S2). It is possible that, in the preactivation IKK complex, NEMO “primes” IKKβ for activation by upstream signaling events, such as polyubiquitin chain binding or phosphorylation by an upstream kinase (29, 30). The dependence of signaling for the IKKβ activation can be overcome by a high IKK concentration, as the NEMO–IKKβ complex can be activated when overexpressed (11). Although, in the postactivation complex, large conformational changes occur to allow the kinase domains to swing away from each other, making room for substrate binding and catalysis. Binding of B14 to the junction of the KD and SDD of IKKβ causes a steric hindrance that impedes optimal contact between KDs in the IKK complex. This structural rearrangement, in turn, blocks insertion of the activation segment of one KD to the active site of another for trans autophosphorylation (Fig. 5 and Fig. S3). B14 binds to both the constitutively active and inactive IKKβ proteins with comparable affinity (Fig. 2*A*). However, its binding to the preactivation complex stops the KD interactions, precluding trans autophosphorylation and activation. B14 can associate with the postactivation complex with little effect on the activity of the kinase (Figs. 1*B* and *2A*) because the binding site of B14 on IKKβ has no overlap with the kinase substrate binding site and the active site (Fig. 4).

## Discussion

### Mechanism of B14-mediated IKKβ inhibition

Our biochemical and computational characterization of B14–IKKβ interaction has identified a B14 binding interface consisting of both the KD and SDD of IKKβ. Although B14 inhibited IκBα phosphorylation in both our autoactivation assay (Fig. 1*A*) and the previously reported *in vivo* experiment in HEK293T cells (18), our *in vitro* kinase inhibition assay clearly shows that B14 does not directly inhibit IκBα phosphorylation by the activated IKKβ (Fig. 1*B*). Because B14 binding attenuates IKKβ dual phosphorylation on Ser-177 and Ser-181 (18), which is required for IKKβ to confer kinase activity, we conclude that B14 specifically targets IKKβ trans autophosphorylation to impede its *in vivo* kinase activity. We propose that the B14 interaction creates steric hindrance to prevent the KDs of IKKβ moving in proximity (Fig. S3), which is a prelude for trans autophosphorylation and kinase activation. The B14 binding leaves some space between the activation segment and the B14 protein molecule. In principle, the activation segment of one IKKβ molecule can still have the chance to contact the active site of another for trans phosphorylation. The KD of IKKβ forms an ensemble with the SDD and ULD, and that restricts the access of the active site of one KD with the activation segment of another. In addition, the dimeric structure of IKKβ further limits the proximity of the active segment and active site of KDs, thereby restricting chances of trans autophosphorylation. It also indicates that, in the presence of the scaffold protein NEMO, the activation of IKKβ requires a well-defined structural architecture of the kinase complex that can allow optimal contact between the kinase domains.

### Impact of IKKβ oligomerization on its activation and B14 inhibition

Although IKKβ stays as a dimer in solution, different types of oligomers have been observed in the crystal structures of both human and *Xenopus* IKKβs (7, 29, 30). In the crystal structures of both *Xenopus* and human IKKβs lacking the NBD, two IKKβ dimers interact on the KD-proximal side of the structure to form a crystal packing tetramer. Because the activation segment of protein kinases has the potential to undergo conformational changes (31), the activation segment of one dimer is able to contact the active site of the other dimer for trans autophosphorylation in the observed crystal packing oligomers (7, 29, 30). In a crystal structure of human IKKβ without the NBD, the two KDs from two different IKKβ dimers form a V-shaped interface to permit trans autophosphorylation (29). Based on the modeling studies, with B14 binding to either IKKβ oligomers observed in the three different crystal structures, it blocks the contact of the KDs in all interfaces (Fig. S3). Under *in vivo* condition, IKKβ needs to be activated in the NEMO–IKKβ complex. However, in the absence of an IKK holocomplex structure, it cannot be concluded that the observed KD interfaces in the crystal structures are relevant for IKKβ trans autophosphorylation *in vivo*. Our computational modeling of the B14–IKKβ interaction in all three crystal packing complexes indicates that the mode of B14 binding to IKKβ is an effective way to block the trans autophosphorylation between neighboring IKKβ dimers (Fig. S3). This is consistent with the experimental observation that co-expression of B14 and IKKβ inhibits the IKKβ trans autoactivation induced by overexpression (Fig. 1*A*).

### Differential binding and inhibition of IKKα and IKKβ by B14

B14 has been shown to have no observable binding to IKKα (18), which indicates that it specifically targets IKKβ to stop the anti-inflammatory canonical NF-κB signaling. Based on the sequence alignment of both human IKKα and IKKβ (Fig. S4*A*), the two important residues of IKKβ that are required for binding to B14, Tyr-259 and Val-410, are conserved in IKKα. However, structural analysis of IKKα has revealed a huge structural variation on the corresponding B14 binding interface in IKKα (Fig. S4*B*), which explains the lack of B14 binding to IKKα. However, the low resolution (4.5 Å) of the IKKα structure limits the reliability of this structural comparison to draw any conclusions (32). More structural *in vitro* and *in vivo* binding studies and kinase assays on B14 and IKKα interactions are needed to address this question in the future.

### Possibility of B14 inhibition of IKKβ activation by TAK1 phosphorylation

Some other studies have shown that the kinase TAK1 can activate IKKβ by directly phosphorylating the activation segment of IKKβ (30). It is not known whether the KD of TAK1 also forms a large multidomain integral structure with the rest of the protein, although it has been demonstrated that the KD alone of TAK1 is crystallizable and can be activated by binding to TAB1 (TAK1-binding protein 1), which is capable of forming an ensemble structure like IKKβ, but TAB1 is not necessary for TAK1 kinase activity (33). A free KD of TAK1, therefore, will have more rotation mobility and accessibility to contact the activation segment of IKKβ for phosphorylation than being held in a larger complex with other domains or proteins. In that case, it is unlikely that the B14 binding will inhibit TAK1 phosphorylation of IKKβ. This implies that phosphorylation by an upstream kinase is not the decisive step for IKKβ activation and that trans autophosphorylation is the main mechanism. Therefore, phosphorylation by TAK1 is merely a priming event that may trigger or augment IKKβ activation. It remains to be tested experimentally whether the presence of B14 indeed has any effect on TAK1 phosphorylation of IKKβ.

### Future structural and in vivo studies to investigate the mechanism of B14-mediated inhibition of IKKβ activation

In this study, we mainly focused on mapping the interaction interface between IKKβ and B14. For a deeper understanding of B14-mediated inhibition of IKKβ activation, structural characterization of both the NEMO–IKKβ and B14–NEMO–IKKβ protein complexes needs to be carried out by X-ray crystallography or cryo-EM. Structural comparisons between both complex structures will provide more mechanistic and molecular insights into IKKβ activation and B14 inhibition. Furthermore, conducting *in vivo* studies to look for NF-κB signaling outcomes is necessary to improve our understanding of B14-mediated IKKβ inhibition.

## Experimental procedures

### Protein expression and purification

Various constructs of human IKKβ WT and the phosphomimic S177E/S181E mutants were designed with an N-terminal polyhistidine tag and a tobacco etch virus protease cutting site engineered between the tag and the protein. Recombinant IKKβ baculoviruses were made in DH10BAC cells, amplified, and used to infect Hi5 insect cells in serum-free medium (Invitrogen and Pharmingen). The cells were cultured in suspension and harvested 48 h post-infection. The recombinant proteins were purified by nickel affinity chromatography, anion exchange, and gel filtration chromatography. The polyhistidine tag was cleaved by the tobacco etch virus protease during protein purification.

All human IκBα and VACV B14 proteins were expressed in *E. coli* using pET28a and pGEX4T3 vectors and purified by their respective affinity tags. GST-KD, GST-KD-ULD, GST-ULD, and GST-ULD-SDD of human IKKβ were also expressed in *E. coli* using pGEX4T3 vectors. The tagged proteins were first purified with GSH or nickel-nitrilotriacetic acid beads, and their expression levels were assessed by SDS-PAGE.

### Transfection, immunoprecipitation, and kinase assay

The HA-B14 construct, Flag-IKKβ, and its mutant constructs were generated in the pcDNA3 vector using conventional PCR. HEK293T or HEK293F cells were transfected with all constructs using Lipofectamine 2000 (Invitrogen). After 24 h, cell extracts were immunoprecipitated with anti-FLAG antibodies bound to agarose beads (M2, Sigma). IKKβ kinase assays were essentially done as described previously (8, 34). The immunoprecipitants were incubated with 2 μm full-length IκBα in 20 mm HEPES (pH 7.5), 10 mm MgCl_2_, 20 mm β-glycerophosphate, 10 mm *p*-nitrophenyl phosphate, 50 mm Na_3_VO_4_, 1 mm DTT, 20 mm ATP, and 1–10 mCi)[γ-^32^P]ATP at 30 °C for 30 min and subjected to SDS-PAGE and autoradiography. Immunoblotting was performed using anti-FLAG (Sigma) or anti-HA (Sigma) antibodies (Upstate, 05-535). For the *in vitro* IKKβ kinase assay, purified recombinant IKKβ-S177ES/182E mutant protein was mixed with varying amounts of recombinant B14 or maltose binding protein as described above.

### Pulldown assays

For GST pulldown, the tagged proteins were purified with GSH or nickel-nitrilotriacetic acid beads, followed by purification with gel filtration using a SuperdexG200 10/300 column (GE Healthcare). The expression levels were assessed by SDS-PAGE. Beads containing estimated equivalent quantities of the GST-tagged proteins were mixed with the purified versions of the interacting partners. The mixtures were incubated at 4 °C for 2 h with continuous rotation on a rocking platform. After centrifugation, the supernatants were removed. The beads were then washed three times with buffer (50 mm Tris-HCl (pH 8.0), 100 mm NaCl, 2 mm β-mercaptoethanol, and 5 mm DTT) and eluted with 25 mm reduced GSH, and the elutions were subjected to SDS-PAGE analysis. All GST pulldown experiments were repeated at least three times for consistency.

For FLAG pulldown, FLAG-tagged human IKKβ and mutant proteins expressed in HEK293F cells were lysed in buffer containing 50 mm Tris-HCl (pH 8.0), 100 mm NaCl, 2 mm β-mercaptoethanol, 5 mm DTT, 2% NP40, and a protease inhibitor mixture. The protein was then mixed with anti-FLAG antibody (Sigma), purified GST-B14 protein, and protein A/G–agarose (Thermo Scientific). The resultant protein complex was eluted by 200 μg/ml FLAG peptide (Sigma) and subsequently used for Western blotting with anti-FLAG or anti-GST antibodies. The image was captured by G:Box imaging systems. All experiments were repeated at least three times.

### Protein molecular docking

Two IKKβ structures (PDB codes 3QA8 (7) and 4E3C (23)) were selected as the receptors, and the B14 structure (PDB code 2VVY (17)) was selected as the ligand. First, the FRODOCK interactive protein–protein docking (22, 23) (http://frodock.chaconlab.org/)^3^ online server was used for a global search to provide the potential binding position information. Because GST pulldown experiments show that the M2 and M4 segments of IKKβ are involved in binging to B14, and Phe-130 of B14 is required for IKKβ interaction, we next used the Cluspro 2.0 protein–protein docking (24, 25) online server (https://cluspro.bu.edu/)^3^ to obtain more accurate complex models with these defined interfaces as restraints. The top ten models were analyzed, and the best-fitting one was chosen as an initial complex model for subsequent optimization with the Rosetta docking 2 (http://rosie.rosettacommons.org/)^3^ online server (26, 27). Local docking was chosen as the docking protocol.

## Author contributions

Q. T., S. C., and G. X. conceptualization; Q. T. and G. X. resources; Q. T., S. C., and G. X. data curation; Q. T. software; Q. T., S. C., and G. X. formal analysis; Q. T. and S. C. validation; Q. T. and G. X. investigation; Q. T. and S. C. visualization; Q. T. and S. C. methodology; Q. T. and G. X. writing-original draft; Q. T., S. C., and G. X. writing-review and editing; G. X. supervision; G. X. funding acquisition; G. X. project administration.

## Supplementary Material

Supporting Information

## Footnotes

3 Please note that the JBC is not responsible for the long-term archiving and maintenance of this site or any other third party–hosted site.

## Footnotes

2 The abbreviations used are:

IKK: inhibitor of κB kinase
NEMO: NF-κB essential modifier
KD: kinase domain
ULD: ubiquitin-like domain
SDD: scaffold and dimerization domain
NBD: NEMO-binding domain
VACV: vaccinia virus
HA: hemagglutinin
GST: glutathione *S*-transferase
HEK: human embryonic kidney.
