# Supplementary material for: Mechanism of vaccinia viral protein B14–mediated inhibition of IκB kinase β activation
Source: J Biol Chem. 2018 May 10;293(26):10344–52. doi: 10.1074/jbc.RA118.002817 (PMC6028965; doi:10.1074/jbc.RA118.002817)
Supplement: Supporting Information [file supp_293_26_10344__index.html]

Mechanism of vaccinia viral protein B14–mediated inhibition of IκB kinase β activation — B14 inhibits IKKβ activation — Supporting Information 

# Mechanism of vaccinia viral protein B14–mediated inhibition of IκB kinase β activation

## Supporting Information

- Mechanism of vaccinia viral protein B14 mediated inhibition of I&#x03BA;B kinase &#x03B2;&#xF020;activation - Supporting Information
