# Supplementary material for: Mechanism of vaccinia viral protein B14–mediated inhibition of IκB kinase β activation
Source: J Biol Chem. 2018 May 10;293(26):10344–52. doi: 10.1074/jbc.RA118.002817 (PMC6028965; doi:10.1074/jbc.RA118.002817)
Supplement: Supporting Information [file supp_RA118.002817_J136546_2_supp_132058_p86149.pdf]

Mechanism of vaccinia viral protein B14 mediated inhibition of IκB kinase β activation

Qingyu Tang[1], Sayan Chakraborty[1], and Guozhou Xu[1,*]

[1]Department of Molecular and Structural Biochemistry, North Carolina State University, Raleigh, NC 27695, USA

[*]To whom correspondence should be addressed. Tel.: 919-515-0835; Email: gxu3@ncsu.edu.

Supporting Information

Materials included: Figure S-1, Figure S-2, Figure S-3, and Figure S-4.

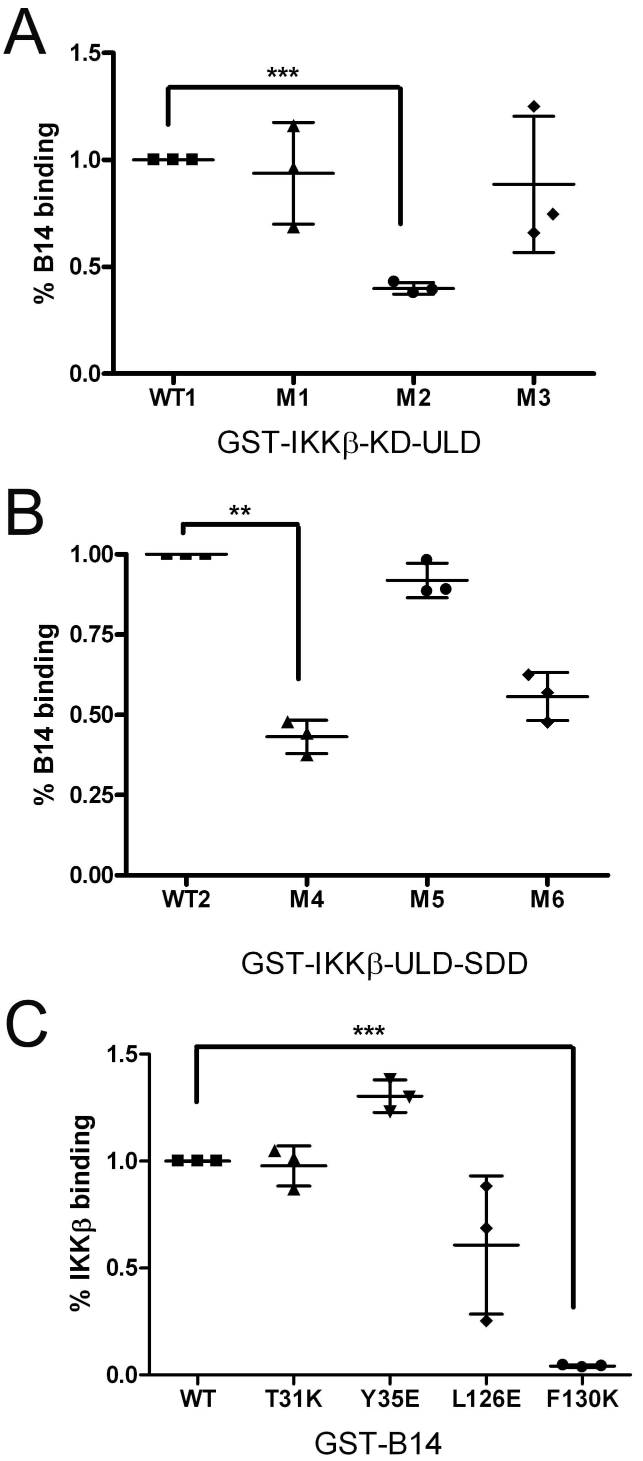

FIGURE S-1. The M2 and M4 segments of IKKβ are required for B14 binding. (*A*) and (*B*) Pull-down of B14 by GST-IKKβ mutants. GST protein alone was used as a negative control. GST-IKKβ-KD-ULD contains residues 1-384 of human IKKβ, and GST-IKKβ-ULD-SDD harbors residues 307-678 of human IKKβ. Both of which are used as the wild type positive controls (WT1 and WT2). In addition, M1

mutants has residues 179-197substituted with GGGGSGGS, M2 with residues 235-260 has been replaced with GGGGSGGS, M3 contains F182K/V183K/L186K mutations, M4 with residues 408-416 replaced with GGGGS, M5 with residues 421-426 substituted with GGGGS, and M6 with residues 577-583 replaced with GGGGS. There are two GST-IKKβ mutant groups, one containing the KD-ULD boundary and the other containing ULD-SDD boundary. The constructs corresponding to WT1, M1, M2 and M3 belong to the KD-ULD group, whereas, WT2, M4, M5 and M6 belong to the ULD-SDD group. The pull down results of each IKKβ mutant construct was compared to the wild type control within each group. To quantify of the relative amount of B14 in the pull down (Fig.3*B*), each band of the GST-IKKβ band was normalized to the same intensity of the GST control band, then the adjusted intensity of the B14 band after subtracting from the GST negative control can be compared to indicate relative binding affinity. We have conducted each experiment three times, and presented with the mean with standard deviation. Triple asterisk shows p value < 0.001 conducted with T test, and double asterisk shows p value < 0.05. (*B*) Pull-down of human IKKβ (1-675, S177E/S181E) by GST-B14 mutants. Each experiment was conducted three times. The quantification of the pull-down results are represented in scattered graphs and presented with standard error of the mean (Fig.3*D*). Triple asterisk shows p value < 0.001 with T test, and double asterisk shows p value < 0.05. All experiments were repeated three times and yielded similar results.

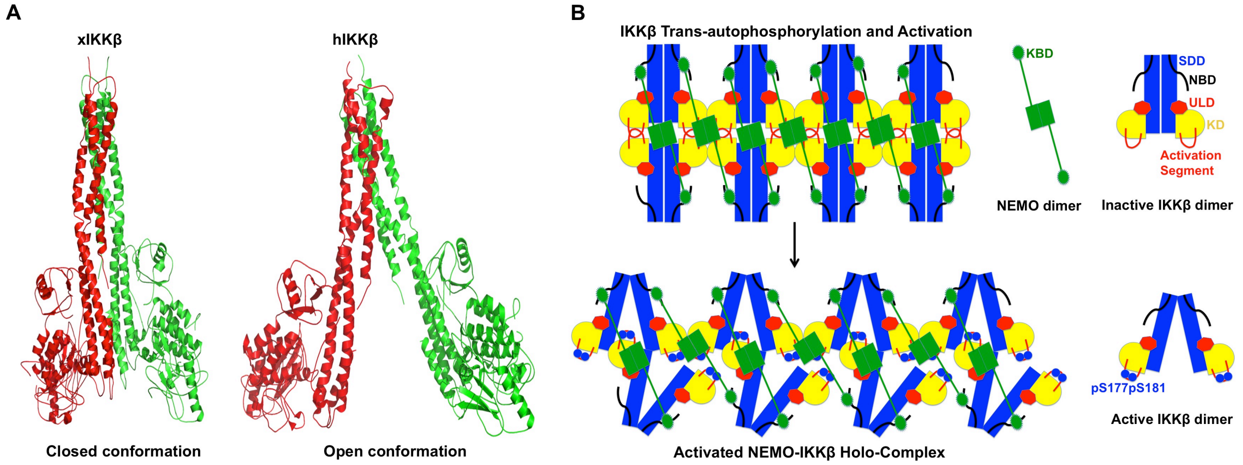

FIGURE S-2. A model for IKKβ trans auto-phosphorylation and activation. (*A*) The *xenopus* IKKβ (xIKKβ, PDB ID: 3QA8) structure adopts a closed conformation and a human IKKβ (hIKKβ, PDB ID: 4E3C) structure stays in an open conformation. (*B*) NEMO crosslinks IKKβ to form a large oligomer. We hypothesize that the IKKβ undergoes at least two transitional stages during its activation, a pre-activation complex and a post-activation (activated) complex. Upon activation by upstream signaling events or by high IKK concentration, the activation segments of the neighboring KDs in the pre-activation complex can contact each other for trans auto-phosphorylation. While in the post-activation complex, large conformational changes occur to allow the kinase domains to swing away from each other, making room for substrate binding and catalysis.

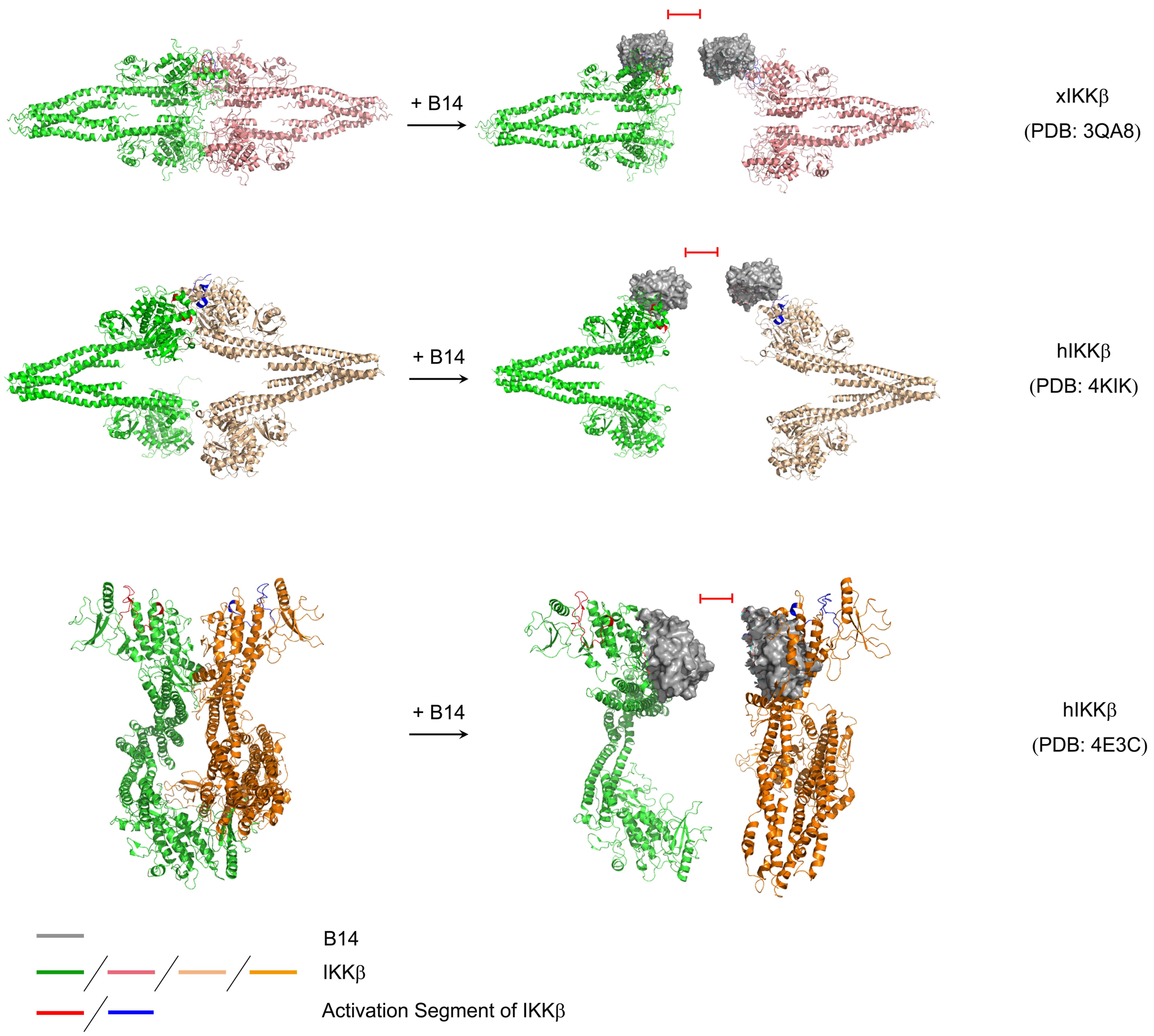

FIGURE S-3. A proposed mechanism of B14-mediated inhibition of IKKβ trans auto-phosphorylation and blocking of subsequent activation. IKKβ has been shown to form three different types of oligomers in the crystal structures. The binding of the B14 would prevent the neighboring activation segments of IKKβ dimers insertion to each other in all three observed IKKβ oligomers. In the models, the IKKβ is shown in ribbon diagram whereas and the B14 is shown in surface representation. The two dimers of IKKβ are shown and the activation segments in one pair of two neighbor subunits are highlighted in either red or blue.

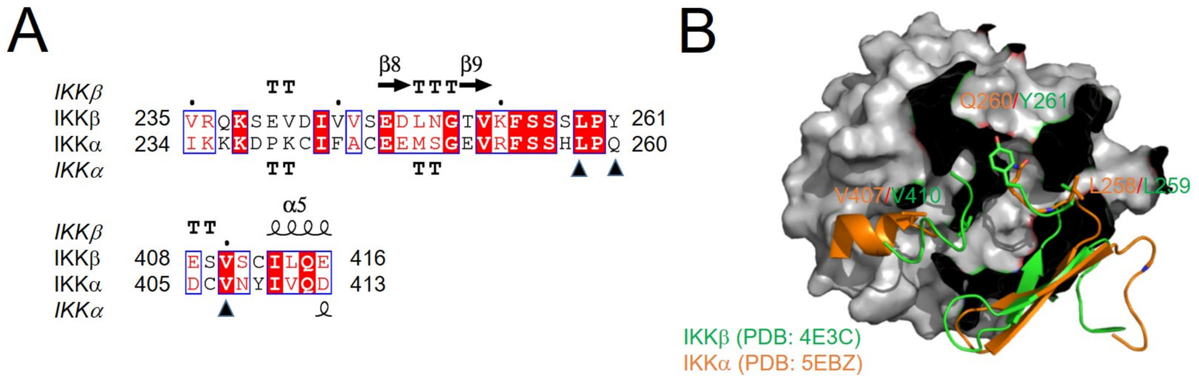

FIGURE S-4. Structural comparision between IKKα and IKKβ. (*A*) Amino acid sequence alignment of the B14 binding region in IKKβ and the corresponding region in IKKα. The secondary structural elements of IKKβ are shown on the top and IKKα at the bottom with α as α-helix, β as β-strand, and TT as turn. The invariant residues are shaded in red boxes and the other less conserved residues are colored in red. The critical B14 interacting residues in IKKβ are indicated with filled black triangles underneath the sequences. (*B*) Superposition of a human IKKβ (PDB ID: 4E3C) and the human IKKα (PDB ID: 5EBZ) structures. The B14 binding region in IKKβ is colored in green, and the corresponding region in IKKα is depicted in orange. The side chains of the potential B14 binding residues are shown in stick representation.
